# Psychometric validation of the PROMIS Fatigue-Short Form 7a in adults with newly diagnosed or recurrent *Mycobacterium avium* complex (MAC) lung disease: the ARISE and ENCORE studies

**DOI:** 10.1186/s41687-025-00944-8

**Published:** 2025-10-16

**Authors:** Kevin C. Mange, Daniel Serrano, Mariam Hassan, Marie-Laure Nevoret, Dayton W. Yuen, Shauna McManus, Lauren Podger, Bryant Barnes, Charles L. Daley

**Affiliations:** 1 Insmed Incorporated, 700 US Highway 202/206, Bridgewater, NJ 08807 USA; 2 The Psychometrics Team, Sheridan, WY USA; 3 OPEN Health, Bethesda, MD USA; 4 OPEN Health, London, UK; 5 National Jewish Health and The University of Colorado School of Medicine, Denver, CO USA

**Keywords:** Fatigue, Lung disease, *Mycobacterium avium* complex, Patient-reported outcome, Psychometric validation

## Abstract

**Background:**

Fatigue symptoms contribute to the burden of *Mycobacterium avium* complex (MAC) lung disease. This study evaluated the psychometric properties of the Patient Reported Outcomes Measurement Information System Short Form v1.0 – Fatigue 7a (PROMIS-F SF-7a) in adults with a new or recurrent diagnosis of MAC lung disease.

**Methods:**

Data from the ARISE (NCT04677543) and ENCORE (NCT04677569) phase 3 trials were analyzed. Modern psychometric methods were employed to confirm the structural validity of the PROMIS-F SF-7a within this context of use. Classical methods were used to confirm the reliability and validity of the PROMIS-F SF-7a within this context of use. Internal consistency (McDonald’s omega, Cronbach’s alpha), test-retest reliability (two-way mixed effects intraclass correlation coefficient [ICC(2,1)]), and known-groups validity across Patient Global Impression of Severity (PGI-S) Fatigue groups were estimated. Convergent validity (Pearson correlations) was assessed by correlating PROMIS-F SF-7a scores with scores on the Exacerbations of Chronic Pulmonary Disease Tool (EXACT), EXACT Respiratory Symptoms (E-RS), St. George Respiratory Questionnaire (SGRQ), and Functional Assessment of Chronic Illness Therapy (FACIT) Fatigue Scale. Meaningful within-patient change (MWPC) thresholds were determined using anchor-based methods.

**Results:**

The baseline sample included 231 patients (99 ARISE, 132 ENCORE). The cross-sectional validation sample comprised 230 patients (excluding 1 ARISE patient with missing item-level PROMIS-F SF-7a data). The longitudinal validation analysis sample comprised all 99 ARISE patients. Modern psychometric methods supported the relevance of all items and a unidimensional unit-weighted sum score for the PROMIS-F SF-7a. The PROMIS-F SF-7a demonstrated strong internal consistency (Cronbach’s alpha: 0.86), test-retest reliability (ICC[2, 1]: 0.76), and convergent validity (FACIT-Fatigue: −0.80, EXACT: 0.56, E-RS: 0.52, SGRQ: 0.66). Known-groups validity was demonstrated across PGI-S Fatigue groups. The MWPC analyses supported a −4.00-point median change from baseline (95% CI: −3.00 to −6.00 points) as the estimated threshold of clinically meaningful within-patient improvement for the PROMIS-F SF-7a.

**Conclusions:**

The PROMIS-F SF-7a is a robust, sensitive, and responsive measure of fatigue in adult patients with a new or recurrent diagnosis of MAC lung disease. It is content and psychometrically valid and appears to have interpretability to assess a threshold of MWPC for fatigue symptoms in this population.

**Trial registration:**

ClinicalTrials.gov. NCT04677543, registered 16 December 2020, https://clinicaltrials.gov/study/NCT04677543. NCT04677569, registered 16 December 2020, https://www.clinicaltrials.gov/study/NCT04677569.

**Supplementary Information:**

The online version contains supplementary material available at 10.1186/s41687-025-00944-8.

## Background

Nontuberculous mycobacteria (NTM) lung disease is an increasingly prevalent condition with symptoms including cough, fatigue, shortness of breath, fever, weight loss, and hemoptysis [1–4]. *Mycobacterium avium* complex (MAC) is the leading cause of NTM lung disease and MAC lung disease is associated with substantial patient and economic burden [1, 5–7].

Patients with NTM lung disease have identified the inclusion of quality of life outcomes in future trials as one of the top priorities for research [8, 9]. Patients have described fatigue as one of the most significant symptoms and are interested in treatments that can reduce fatigue [8, 9]. The impact of fatigue on patients’ daily lives is substantial: patients have reported that it can be sudden, unpredictable, and debilitating and makes it difficult for them to perform personal care tasks, complete their job duties, or participate in activities that they previously enjoyed [8–10]. Patient-reported outcome (PRO) instruments are important tools for incorporating patient experience data into medical product development and regulatory decision-making [11]. However, there are currently no validated PRO instruments to evaluate fatigue symptoms in patients with MAC lung disease [12]. In a 2019 workshop convened by the United States Food and Drug Administration (FDA), clinical experts identified the lack of an NTM lung disease-specific PRO instrument as a key challenge in designing clinical trials for NTM lung disease treatments [12].

The Patient Reported Outcomes Measurement Information System Short Form v1.0 – Fatigue 7a (PROMIS-F SF-7a) instrument has been validated to measure fatigue symptoms in adult patients with chronic obstructive pulmonary disease and other conditions [13–16], but has not yet been validated for use in MAC lung disease. Therefore, several studies were conducted to understand whether the PROMIS-F SF-7a is relevant, comprehensible, and appropriate in patients with MAC lung disease, in keeping with the FDA’s guidance for patient-focused drug development [11, 17, 18]. First, a qualitative study was conducted to collect data to support the selection or development of PRO instruments as fit-for-purpose to measure symptoms in patients experiencing MAC lung disease [19]. In the concept elicitation phase, participants identified respiratory and fatigue symptoms as the most prevalent and bothersome to patients with MAC lung disease, and the most prevalent and important fatigue symptoms (e.g., tiredness, lack of energy, low stamina) are covered by existing PROMIS-F SF-7a items [19]. During cognitive interviews, participants confirmed the overall relevance, comprehensibility, and appropriateness of the instrument among patients with MAC lung disease, and the recall period, response options, and concept attributes were suitable and meaningful to most patients [19].

Given these qualitative findings, the next step was to quantitatively assess the psychometric properties of the PROMIS-F SF-7a in this population in order to support its use in future MAC lung disease treatment research and regulatory decisions. The objective of the psychometric validation analysis was to confirm the dimensionality of the PROMIS-F SF-7a and evaluate the instrument’s scoring, reliability, validity, and responsiveness in adults with a new or recurrent diagnosis of MAC lung disease who had not initiated antibiotics for their current MAC infection.

## Methods

### Study design and participants

**Fig. 1:**
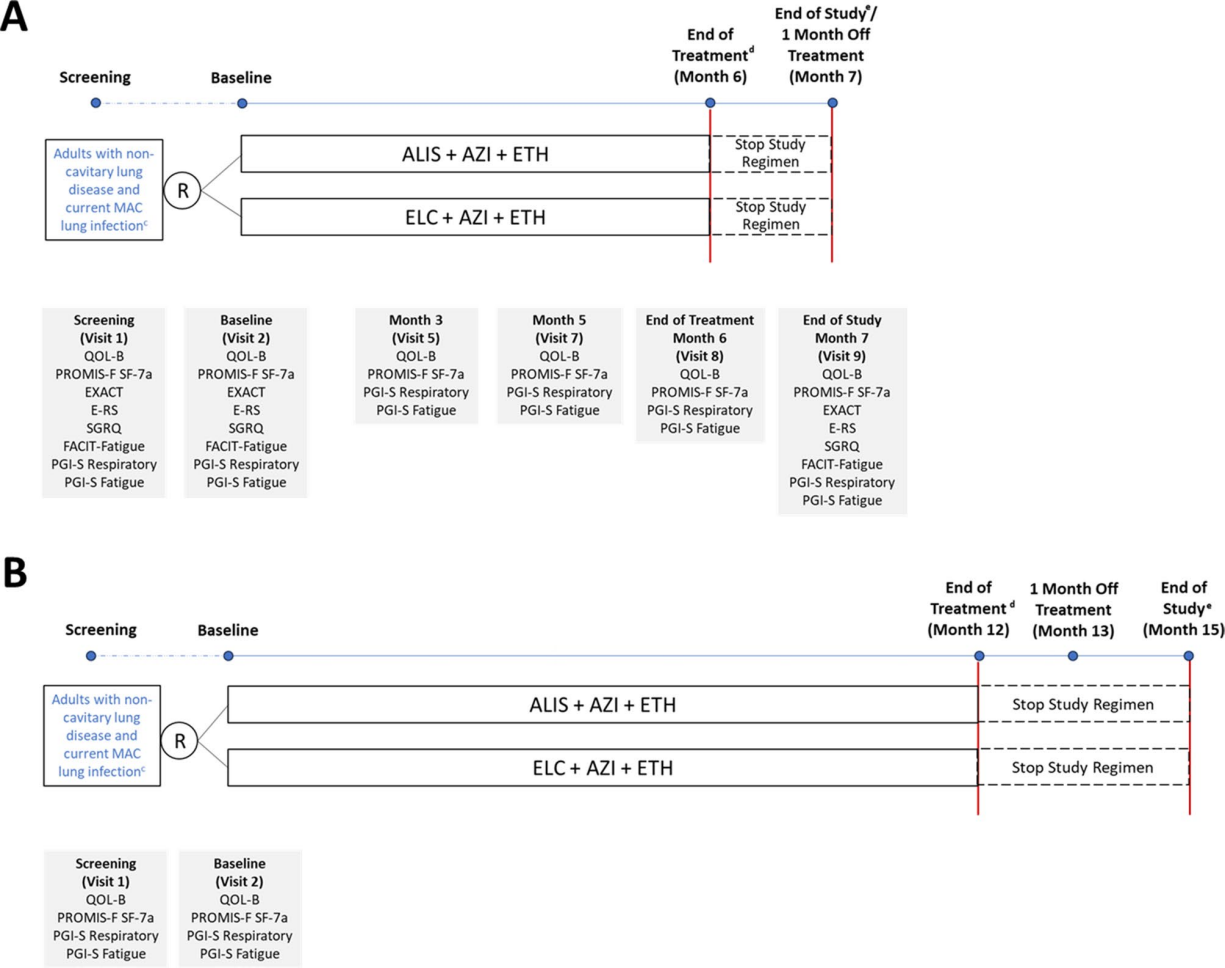
Study Design and Assessments for (**A**) ARISE^a^ and (**B**) ENCORE^b^. ^a^ In ARISE, treatment was administered continuously for 6 months, followed by PRO evaluations and end of study evaluation at month 7 (1 month off treatment). ^b^ In ENCORE, treatment is administered continuously for 12 months, followed by PRO evaluations at month 13 (1 month off treatment) and month 15 (end of study). ^c^ Eligible patients were adults with non-cavitary lung disease who had a current MAC lung infection diagnosis (initial [first] or subsequent [second or third] diagnosis) and had not started treatment for their current MAC lung infection. ^d^ End of treatment is defined as the date of the last dose of ALIS/ELC, AZI and/or ETH, whichever study drug dose is taken last. ^e^ End of study is defined as the date of the last study visit completed by the participant. Abbreviations: ALIS, amikacin liposome inhalation suspension; AZI, azithromycin; ELC, empty liposome control; E-RS, Exacerbations of Chronic Pulmonary Disease Tool Respiratory Symptoms; ETH, ethambutol; EXACT, Exacerbations of Chronic Pulmonary Disease Tool; FACIT-Fatigue, Functional Assessment of Chronic Illness Therapy Fatigue Scale; MAC, *Mycobacterium avium* complex; PGI-S, Patient Global Impression of Severity; PRO, patient-reported outcome; PROMIS-F SF-7a, Patient-Reported Outcomes Measurement Information System Short Form v1.0 – Fatigue 7a; QOL-B, Quality of Life Bronchiectasis; R, randomization; SGRQ, St. George Respiratory Questionnaire

For this validation analysis, we used blinded screening, baseline, and month 7 (1-month off treatment) data from two multicenter randomized controlled phase 3 trials: ARISE (NCT04677543) [20] and ENCORE (NCT04677569) [21] (Fig. 1). The two studies were conducted concurrently and had identical eligibility criteria (Supplementary Table 1). Briefly, eligible patients were adults (aged ≥ 18 years) with non-cavitary lung disease who had a current MAC lung infection diagnosis (initial [first] or subsequent [second or third] diagnosis) and had not started treatment for their current MAC lung infection. Candidates were randomly assigned to either study at the time of informed consent until ARISE was fully enrolled. In both trials, study participants were randomized in a 1:1 fashion to amikacin liposome inhalation suspension (ALIS) 590 mg or empty liposome control once daily, in combination with a background regimen of azithromycin 250 mg and ethambutol 15 mg/kg tablets once daily.

The primary objective of ARISE was to generate evidence in support of the validation of the PROMIS-F SF-7a and the Quality of Life Questionnaire–Bronchiectasis respiratory domain for use in adult patients with MAC lung disease [20]. The primary objective of ENCORE is to evaluate the efficacy of ALIS plus background regimen compared to empty liposome control plus background regimen on patient-reported outcomes following completion of treatment [21]. Both studies were conducted in accordance with the Declaration of Helsinki. Local institutional review boards or independent ethics committees approved the study protocols. All patients provided written or electronic informed consent.

### PRO assessments

The PROMIS-F SF-7a is a self-administered questionnaire derived from the PROMIS Fatigue item banks. The item banks cover a range of self-reported fatigue symptoms over the past 7 days, from mild subjective feelings of tiredness to an overwhelming, debilitating, and sustained sense of exhaustion [13]. Fatigue is divided into the experience of fatigue (frequency, duration, and intensity) and the impact of fatigue on physical, mental, and social activities [22, 23]. Response options are on a 5-point Likert scale, ranging from 1 (never) to 5 (always). The PROMIS-F SF-7a raw score is the sum of item responses, ranging from 7 (no impairment) to 35 (worst fatigue symptoms), with higher scores indicating greater fatigue [22]. Previous studies have supported the unidimensionality of the PROMIS-F SF-7a [13]. For the purposes of validation, we analyzed raw (untransformed) PROMIS-F SF-7a scores.

The ARISE study included multiple PROs to support validation of the PROMIS-F SF-7a. First, the Patient Global Impression of Severity (PGI-S) Fatigue is a simple categorical rating of fatigue symptom severity over the past week on a scale from 1 (not at all) to 5 (extremely severe) [24, 25]. The PGI-S Fatigue also served as an anchor for the analysis of meaningful within-patient change (MWPC) in PROMIS-F SF-7a scores because it assesses the same concept of interest (fatigue) and has the same recall period (past week) [26]. Other validating PROs were total scores from the Exacerbations of Chronic Pulmonary Disease Tool (EXACT) [27–29], EXACT Respiratory Symptoms (E-RS) [30, 31], St. George Respiratory Questionnaire (SGRQ) [32], and Functional Assessment of Chronic Illness Therapy (FACIT) Fatigue Scale [33].

The EXACT PRO is a daily diary composed of 14 items assessing dyspnea, cough, sputum production, chest symptoms, difficulty expectorating, fatigue, sleep disturbance, and fear or concern. Each item is assessed on a 5- or 6-point ordinal scale and summed to yield a total score that is converted to a 0–100 scale, with higher values indicating a more severe condition [29]. The E-RS is an 11-item subset of the EXACT (items 1–11) that assesses exacerbations of respiratory symptoms; it yields a total score ranging from 0 to 40, with higher values indicating a more severe condition [30]. The SGRQ consists of 50 items with 76 weighted responses grouped into a set of 17 questions, some of which allow multiple responses. Part 1 (symptom component) evaluates symptom frequency and severity with a 1-month recall using 4- or 5-point scales. Part 2 (activity and impact components) addresses individuals’ current state using primarily true/false response options. SGRQ total scores range from 0 to 100, with higher scores indicating higher severity [32]. The FACIT-Fatigue Scale contains 13 items measuring an individual’s level of fatigue during their usual daily activities over the past week on a Likert scale from 4 (not at all fatigued) to 0 (very much fatigued). The FACIT-Fatigue score ranges from 0 to 52, with higher scores indicating lower fatigue.

In ARISE, participants completed PRO assessments at pre-specified in-clinic visits (Fig. 1). For both studies, the PRO instruments were provided in electronic format on a computer tablet and conducted at approximately the same time of day at the specified timepoints. Trial participants completed the PRO assessments prior to other study assessments (e.g., physical examination, vital signs, laboratory assessments, audiology) and administration of study drugs.

### Statistical analysis

#### Analysis samples

The cross-sectional validation sample comprised data from 230 patients. This included screening and baseline data from 98 patients in ARISE who provided item-level PROMIS-F SF-7a data as well as baseline data from the first 132 patients randomized into ENCORE. Item-level PROMIS-F SF-7a data were missing for one patient in the ARISE study.

The longitudinal validation analysis sample comprised all 99 patients enrolled in ARISE who completed baseline and 7-month follow-up (i.e., end of study/1 month off treatment) visits. We imputed PROMIS-F SF-7a raw scores for one patient in ARISE who was missing item-level data (Supplementary Material). PROMIS-F SF-7a T-scores were available for 91 patients in ARISE; missing data were not imputed.

#### Cross-sectional validation

##### Item-level descriptive assessments

Response patterns for PROMIS-F SF-7a items were generated to test for floor and ceiling effects (i.e., lowest and/or highest response categories endorsed by > 20% of participants) and response category sparseness (i.e., categories not endorsed or endorsed by < 10% of participants). To understand the pattern of item associations, inter-item correlations were estimated using polychoric correlations [34].

##### Modern psychometric methods

We employed modern psychometric methods to confirm the structural validity of the PROMIS-F SF-7a in the new or recurrent MAC lung disease context of use. First, we conducted item exploratory factor analysis (iEFA) to identify and define domains measured by the PROMIS-F SF-7a items (i.e., what concepts are assessed by which items in a questionnaire). The iEFA models were estimated using Samejima’s graded response models (GRM) [35], which is the analog of a multivariate proportional odds model. We determined the number of factors required to adequately characterize PROMIS-F SF-7a data using model fit indices: the C2-based $\:{{\upchi\:}}^{2}$ and root mean squared error of approximation (RMSEA) and corresponding 90% confidence interval (CI). Two fit conditions were considered for C2-based statistics: the first was exact fit, corresponding to a null $\:{{\upchi\:}}^{2}$p-value; the second was the test of close fit, corresponding to an RMSEA lower confidence limit less than 0.05 [36]. In addition, we employed the Tucker-Lewis Index (TLI) and comparative fit index (CFI) in model fit evaluations, with values of 0.95 indicating good fit [37].

We used item response theory (IRT) models to refine structural validity evidence. The IRT models, parameterized with Samejima’s GRM, tested the quality and precision of PROMIS-F SF-7a items as well as item redundancy and bias. In addition, we employed dimensionality and scoring statistics to empirically determine the essential dimensionality of PROMIS-F SF-7a scores [38]. To assess essential dimensionality, we compared multidimensional IRT and bifactor models. Model fit indices of C2-based $\:{{\upchi\:}}^{2}$ and RMSEA, TFI, and CFI were employed to determine which model explained the data best. Dimensionality statistics used to evaluate essential dimensionality and scoring included the explained common variance (ECV) [39], H statistic, and McDonald’s omega ratios [40]. The ECV can be thought of as the proportion of variance of the item responses that can be attributed to the total domain. H is the correlation between the domain(s) (either subdomains or total) and corresponding domain scores. The omega ratio is the ratio of omega statistics reflecting the variance explained by the general factor (omega hierarchical) to the variance explained by the subdomains (omega). Essential unidimensionality of the PROMIS-F SF-7a data (i.e., the data can be accurately characterized by a unidimensional IRT model) was supported if ECV values were greater than or equal to 0.9. Essential unidimensionality of the PROMIS-F SF-7a unit-weighted score was supported if H values exceeded 0.80, and the ratio of all modellable sources explaining variance in a unit-weighted total score ($\:{\upomega\:}$) to that variance in a unit-weighted total score explained by a total domain ($\:{{\upomega\:}}_{\text{H}}$) (i.e., $\:{{\upomega\:}}_{\text{H}}/{\upomega\:}$) was equal to or greater than 0.80. Such an omega ratio for a total domain obtained from a bifactor model would indicate that 80% of the variance in a unit-weighted total score would be accounted for by the total domain, empirically justifying an essentially unidimensional unit-weighted total score [41].

Once the final IRT domain structure was established, we evaluated slopes and intercepts for each item in order to identify and retain items with strong measurement properties and, if applicable, eliminate items with poor measurement properties. For the remaining items qualified for analysis, we converted item parameters to item response functions (IRFs), which are the predicted probabilities for endorsement of each PROMIS-F SF-7a item implied by the model. The steepness of the slope characterizes the strength of association between the latent variable and the item response, with steeper slopes demonstrating that the item strongly measures the domain, and flat IRFs reflecting poorly performing items.

We evaluated item redundancy by identifying local dependence among items using Chen and Thissen’s G^2^ statistic [42], with any item demonstrating a significant marginal χ^2^ or a G^2^ value greater than 3 indicating problematic local dependence. Differential item functioning (DIF) analysis was conducted using the Langer-improved Wald-2 DIF sweep procedure [43] to determine whether younger versus older participants (defined on median split of age distribution), male versus female participants, and participants with an initial versus subsequent MAC lung infection responded to the items differentially. Inferential tests employed in local dependence and DIF analyses were multiplicity-adjusted using the Benjamini-Hochberg false discovery rate adjustment [44]. Additional details on the Wald-2 DIF sweep procedure are available in the Supplementary Material.

##### Reliability and validity

We used classical test theory methods of reliability and validity to assess the PROMIS-F SF-7a score properties. Internal consistency and known-groups validity were analyzed using baseline data from the full cross-sectional validation sample. Test-retest reliability was analyzed using screening to baseline data from the ARISE patients only. Convergent validity was estimated using baseline data from the ARISE patients only.

Internal consistency was estimated using the McDonald’s omega statistic and Cronbach’s alpha [34]. We supplemented these estimates with item-to-total correlations estimated via polyserial correlations [45]. Cronbach’s alpha and omega estimates of 0.7 or greater and item-to-total correlations of 0.4 or greater were considered as evidence of acceptable internal consistency [46–48].

Test-retest reliability was estimated in a stable subgroup of patients reporting no change (i.e., change score of zero) in PGI-S Fatigue between screening and baseline (up to 70-day retest interval). Test-retest reliability correlations were based on the two-way mixed effects intraclass correlation coefficient (ICC[2,1]) [49]. ICC estimates of 0.7 or greater demonstrated satisfactory test-retest reliability [46].

Convergent validity was assessed by correlating the PROMIS-F SF-7a and PRO measures that were hypothesized to assess the same or similar concepts. Specifically, we calculated correlations between the PROMIS-F SF-7a and the EXACT, E-RS scale, SGRQ, and FACIT-Fatigue scores at baseline. Pearson correlations of 0.4 or greater met the prespecified criterion for acceptable convergent validity [46]. We expected a larger magnitude of correlation with the FACIT-Fatigue, given it is another measure of fatigue, and comparatively smaller magnitude of correlations with the EXACT, E-RS scale, and SGRQ, which are more distal measures of general and respiratory-specific symptoms and respiratory health-related quality of life.

Known-groups validity was assessed by comparing PROMIS-F SF-7a scores across PGI-S Fatigue severity groups. We fit a linear model to the baseline PROMIS-F SF-7a scores and compared least squares means for the low-severity PGI-S Fatigue group (reference) against each of the other PGI-S Fatigue severity groups (effect) in pair-wise contrasts. Acceptable known-groups validity was achieved if a preponderance of the known-effect groups had ordered means and corresponding effect sizes greater than 5%.

#### Longitudinal validation

##### Responsiveness

To assess whether the PROMIS-F SF-7a was sufficiently sensitive to reflect clinically meaningful changes within patients over time, we estimated anchor-based thresholds of MWPC using ARISE data only. PROMIS-F SF-7a mean and median change scores were computed between baseline and end of study (i.e., 1-month off treatment [month 7]) in the longitudinal validation analysis sample (*N* = 99 ARISE patients for raw scores; *N* = 91 patients for T-scores). We used the anchor-based approach and supplemented with empirical cumulative distribution function (eCDF) curves in accordance with FDA guidance on incorporating clinical outcome assessments (COAs) into study endpoints [26]. Anchor-based methods are sufficient to identify meaningful score differences from patients’ categorizations of their change in symptom severity, whereas distribution-based methods alone are insufficient as they do not directly consider the patient voice [26].

The PGI-S Fatigue anchor scale has response options of ‘not at all’ (1), ‘mildly’ (2), ‘moderately’ (3), ‘very’ (4), or ‘extremely severe’ (5). We characterized the MWPC thresholds via point estimates obtained from the median change score associated with corresponding PGI-S Fatigue change groups (i.e., improved ≥ 2 categories, improved 1 category, maintained, deteriorated 1 category, deteriorated ≥ 2 categories). In line with FDA guidance on COA-based endpoints, improvement of 1 category on the PGI-S Fatigue was used to define the meaningful improvement anchor group, from which we estimated improvement thresholds [26]. We generated an eCDF curve to visualize the degree of separation (if any) between the anchor change groups at the location of the point estimate (i.e., median change score).

## Results

### Study population

Within the overall baseline sample, which included 132 ENCORE patients and all 99 ARISE patients (including the 1 patient who was excluded from the cross-sectional validation sample, but included in the longitudinal validation sample), 68% of participants were aged 65 years or older, 81% were female, and 73% were White (Table 1). Patients were enrolled between November 12, 2020, and March 8, 2023.

**Table 1:** Sample characteristics

	Baseline Sample (*N* = 231)^a^
Age group, n (%)	
< 65 years	75 (32.6)
≥ 65 years	156 (67.8)
Sex, n (%)	
Female	186 (80.9)
Male	45 (19.6)
Race, n (%)	
American Indian or Alaska Native	0 (0.0)
Asian	55 (23.9)
Black or African American	1 (0.4)
Native Hawaiian or Other Pacific Islander	0 (0.0)
White	168 (73.0)
Other	0 (0.0)
Multiple	0 (0.0)
Not reported	6 (2.6)
Unknown	1 (0.4)
Ethnicity, n (%)	
Hispanic or Latino	11 (4.8)
Not Hispanic or Latino	214 (93.0)
Not reported	6 (2.6)
Weight at baseline, mean (min-max), kg	60.16 (36.0-139.0)
Height at baseline, mean (min-max), cm	164.79 (132.1-195.6)
BMI at baseline, mean (min-max), kg/m^2^	22.02 (13.6–42.0)
FEV_1_, mean (min-max), L	1.97 (0.55–4.22)

^a^ The baseline sample description is based on 231 patients: *n* = 99 ARISE patients (including the 1 patient who was excluded from the cross-sectional validation sample, but included in the longitudinal validation sample) and *n* = 132 ENCORE patients. Due to rounding, percentages may total more than 100%

Abbreviations: BMI, body mass index; FEV_1_, forced expiratory volume in 1 s

### Cross-sectional validation

#### Item-level descriptive assessments

No items demonstrated item response missingness. Although item endorsement suggested the presence of floor effects for 3 out of 7 items (43%; Supplementary Table 2), the items’ other response categories were well endorsed by patients, demonstrating the items’ utility. Therefore, we retained all items due to qualitative evidence of relevance. There was no evidence of ceiling effects. Response category sparseness was demonstrated for 6 out of 7 items for the ‘always’ response category, but these instances of spareness were indicative of the baseline level of symptom severity in the sample per the predefined inclusion criteria.

Overall, 22 of the 28 unique inter-item correlations (79%) met or exceeded *r* = 0.4 (Supplementary Table 3). A single item, ‘enough energy to exercise,’ accounted for the inter-item correlations falling below the pre-specified acceptability criterion, with correlations across the other 6 items ranging from *r* = 0.13 to 0.28. The results indicate that the PROMIS-F SF-7a is largely composed of items expected to define a monolithic score, with varying strengths of association and no evidence of redundant items.

#### Modern psychometric methods

**Fig. 2:**
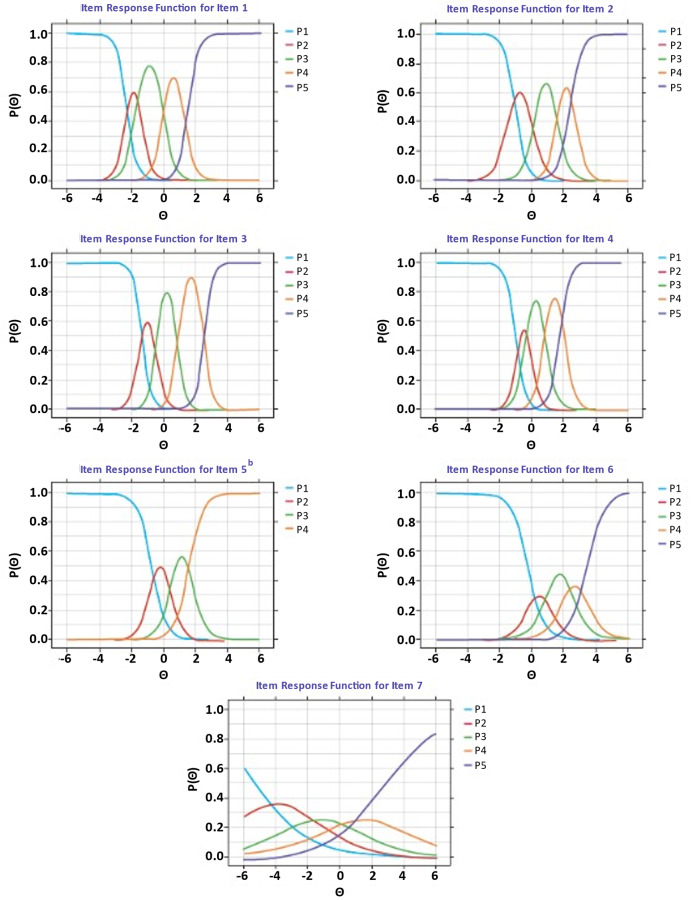
Modern psychometric methods: IRFs for PROMIS-F SF-7a^a^. ^a^ Used data from the cross-sectional validation sample (*N* = 230 patients). Each curve represents the probability of endorsing a response category: P1 = probability of endorsing response category 1 (‘never’), P2 = probability of endorsing response category 2 (‘rarely’), etc. ^b^ No participant endorsed the ‘always’ response category for PROMIS-F SF-7a item 5. Abbreviations: IRF, item response function; PROMIS-F SF-7a, Patient-Reported Outcomes Measurement Information System Short Form v1.0 – Fatigue 7a

Modern psychometric methods supported the relevance of all items and a unidimensional unit-weighted sum score for the PROMIS-F SF-7a. For the iEFA, the unidimensional model fit the data very closely (RMSEA, 0.073; 90% CI, 0.038–0.108), and the omega statistic (0.92) exceeded the threshold of 0.80 for supporting a unit-weighted sum score. For the unidimensional IRT model, the lower limit of the RMSEA CI was less than 0.05, satisfying the test of close fit; the TLI (0.98) and CFI (0.97) both approached their maximum values of 1, indicating a very strong fit; and the omega statistic demonstrated that 92% of the variance in a unit-weighted total score was explained by the unidimensional factor of fatigue severity. For the test of exact fit, the $\:{{\upchi\:}}^{2}$p-value was 0.005. Despite the significant χ², other fit indices (ECV, 1.00; omega ratio, 1.00; H statistic, 0.95) and prior PROMIS-F SF-7a validation support unidimensionality. Slopes were numerically large for all items except item 7, which had a low but acceptable slope estimate (Fig. 2), demonstrating that the PROMIS-F SF-7a items discriminate well and that the discrimination is well ordered and distributed.

After adjusting for multiple comparisons using the Benjamini-Hochberg false discovery rate adjustment, there was no significant local dependence among items. In the first stage of the DIF sweep, all items met criteria for anchor items (i.e., no item presented with significant potential DIF between the reference and focal groups after multiplicity adjustment; see Supplemental Tables 5–7). Therefore, there was no evidence of systematic bias between groups in the PROMIS-F SF-7a items.

#### Reliability and validity

The PROMIS-F SF-7a demonstrated strong internal consistency (Table 2), evidenced by a McDonald’s omega of 0.87 and a Cronbach’s alpha of 0.86. Corrected item-to-total correlations further supported a determination of acceptable internal consistency, whereby no correlations fell below the prespecified success criterion of |r| >0.4. Test-retest reliability was estimated among 55 patients whose PGI-S Fatigue score was identical at screening and baseline. The mean interval between the two PROMIS-F SF-7a assessments was 58.31 days (median 60.00 days). This interval was longer than typical of test-retest reliability testing because patients could not be randomized (Baseline visit) until final MAC culture results were available from sputum samples collected at screening, which may take up to 8 weeks due to the slow-growing nature of NTM. A shorter interval subset analysis was not feasible due to the small sample size. The ICC[2,1] estimate was 0.76, indicating acceptable test-retest reliability over a retest interval of up to 70 days.

**Table 2:** Reliability: PROMIS-F SF-7a internal consistency estimates at baseline

PROMIS-F SF-7a Items	McDonald’s omega (95% CI)	Cronbach’s alpha (95% CI)	Item-Total Polyserial Correlation	Cronbach’s alpha If Item Is Dropped
Total score	0.87 (0.84, 0.89)	0.86 (0.83, 0.89)		
Feel tired			0.87	0.83
Extreme exhaustion			0.84	0.84
Run out of energy			0.89	0.83
Fatigue limit work			0.89	0.83
Too tired to think			0.85	0.84
Too tired to bathe			0.81	0.85
Enough energy to exercise			0.46	0.90

Abbreviations: CI, confidence interval; PROMIS-F SF-7a, Patient-Reported Outcomes Measurement Information System Short Form v1.0 – Fatigue 7a

Convergent validity was also supported. As expected, we observed the strongest association between the PROMIS-F SF-7a and the FACIT-Fatigue (−0.80). The moderate positive correlations between the PROMIS-F SF-7a and the EXACT (0.56) and E-RS (0.52) and the moderate-to-strong positive correlation (0.66) for the SGRQ suggest that increasing fatigue levels correspond to diminished respiratory health-related quality of life.

Known-groups validity was demonstrated across the PGI-S Fatigue severity groups (Table 3). Patients who responded ‘not at all’ on the PGI-S-Fatigue had significantly lower PROMIS-F SF-7a scores (i.e., less fatigue) at baseline compared with those who selected any of the other responses (*P* < 0.001). Specifically, a sequentially ordered higher mean PROMIS-F SF-7a score (i.e., more fatigue) was observed at each increased PGI-S Fatigue severity level (i.e., ‘mildly,’ ‘moderately,’ ‘very,’ and ‘extremely’), as expected.

**Table 3:** Known-groups validity at baseline (*N* = 230 patients)

PGI-S Fatigue Reference Category	PGI-S Fatigue Effect Category	*N*	LSM Estimate (95% CI)	LSM Contrast	*P*-value	Semi-Partial Omega Squared (95% CI)
Not at all		21	11.81 (10.33, 13.29)			
	Mildly	88	15.67 (14.95, 16.40)	-3.86	< 0.001	0.08 (0.03, 0.16)
	Moderately	83	20.64 (19.90, 21.38)	-8.83	< 0.001	0.32 (0.23, 0.41)
	Very	34	24.41 (23.25, 25.58)	-12.60	< 0.001	0.43 (0.34, 0.51)
	Extremely	4	25.75 (22.35, 29.15)	-13.94	< 0.001	0.19 (0.11, 0.28)

Abbreviations: CI, confidence interval; LSM, least squares means; PGI-S, Patient Global Impression of Severity

### Longitudinal validation

#### Responsiveness

Prior to the MWPC analysis, we calculated Pearson correlations between PROMIS-F SF-7a and PGI-S Fatigue change scores at end of study (1-month off treatment). A correlation of 0.68 indicated that the PGI-S Fatigue was a suitable anchor for use in these analyses [50]. 

**Fig. 3:**
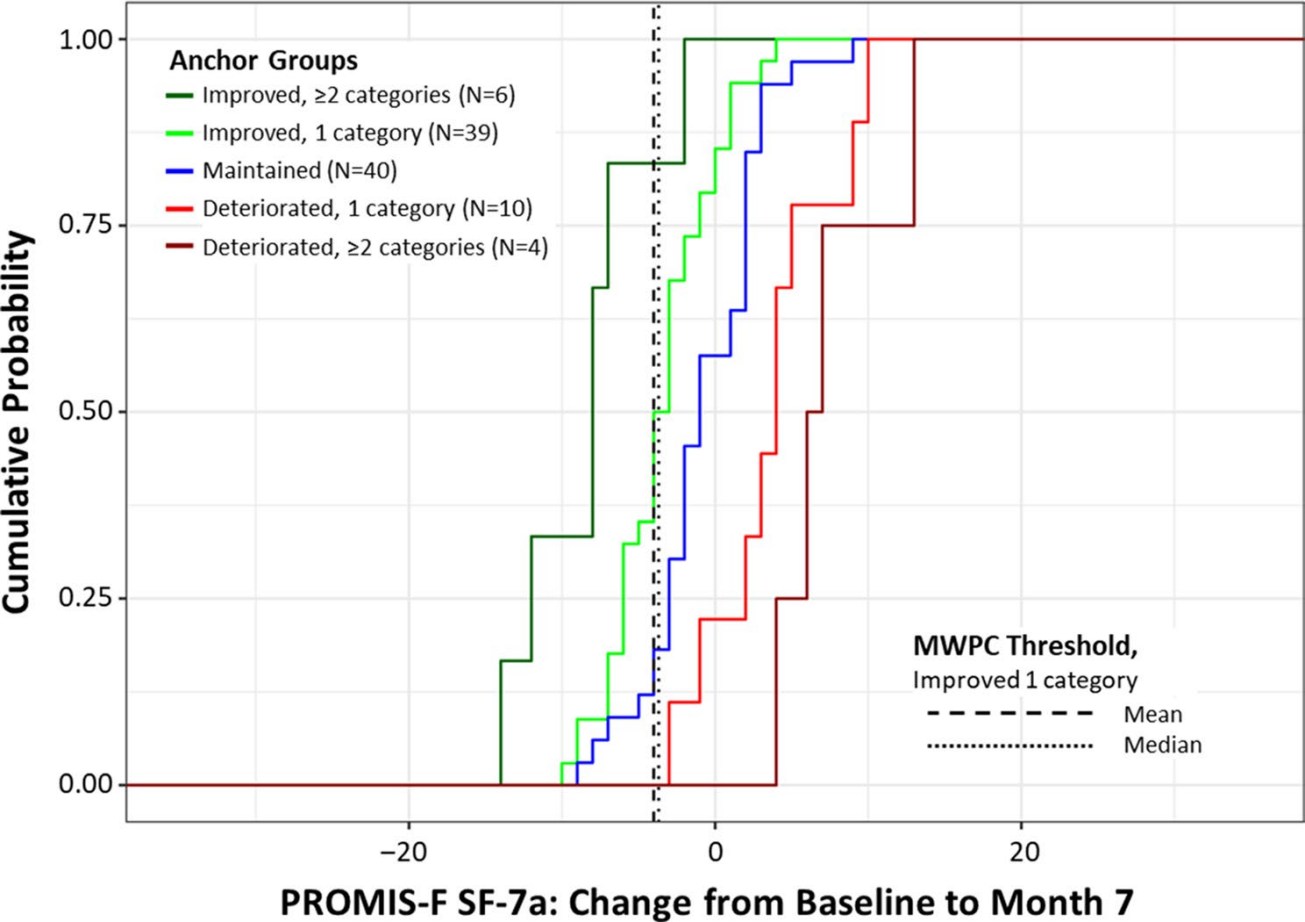
eCDF of PROMIS-F SF-7a change from baseline to end of study by anchor group (*N* = 99 ARISE patients)^a^. ^a^ Used data from the longitudinal validation sample. End of study: 1 month off treatment (month 7). Abbreviations: eCDF, empirical cumulative distribution function; PROMIS-F SF-7a, Patient-Reported Outcomes Measurement Information System Short Form v1.0 – Fatigue 7a

The MWPC analyses supported a −4.00-point median change from baseline (95% CI: −3.00 to −6.00 points) in raw scores as the estimated threshold and plausible range of clinically meaningful within-patient improvement for the PROMIS-F SF-7a (Table 4). The approximate equivalent 1-category improved median threshold for T-scores was −6.20 points. The eCDF curves showed clear and consistent separation between the improved and maintained anchor groups across all PROMIS-F SF-7a score changes, including the −4.00 threshold (Fig. 3). The cumulative percentage of patients in the 1-category improved anchor group achieving the meaningful improvement threshold (−4.00) at end of study (Month 7) was approximately 39% compared with approximately 10% in the maintained anchor group.

**Table 4:** Anchor-based mean and median change from baseline in the PROMIS-F SF-7a raw score at end of study (*N* = 99 ARISE patients)^a^

Anchor Group	*N*	Thresholds by Anchor Group	
		Mean (95% CI)	Median (95% CI)
Improved, 2 + categories	6	-8.50 (-12.89, -4.11)	-8.00 (-14.00, -7.00)
Improved, 1 category	39	-3.69 (-4.77, -2.61)	-4.00 (-6.00, -3.00)
Maintained	40	-0.50 (-1.63, 0.63)	-1.00 (-2.00, 2.00)
Deteriorated, 1 category	10	3.40 (0.51, 6.29)	3.00 (-1.00, 5.00)
Deteriorated, 2 + categories	4	7.50 (NA)^b^	6.00 (NA)^b^

^a^ Estimated using data from the longitudinal validation analysis sample. End of study: 1 month off treatment (month 7)

^b^ Sample size did not permit calculation of confidence intervals

Abbreviations: CI, confidence interval; NA, not applicable; PROMIS-F SF-7a, Patient-Reported Outcomes Measurement Information System Short Form v1.0 – Fatigue 7a

## Discussion

Fatigue is a disabling symptom in chronic respiratory diseases but is insufficiently studied and poorly understood [51]. Its etiology and manifestations are complex and multifactorial, with events such as infectious exacerbations and treatments further perpetuating already very dynamic experiences of fatigue [51]. This is the first psychometric validation analysis of a PRO instrument to assess fatigue in a population with MAC lung disease as part of a clinical trial. The results show that the PROMIS-F SF-7a is a robust, sensitive, and responsive PRO measure of fatigue in adult patients with a new or recurrent diagnosis of MAC lung disease. The PROMIS-F SF-7a is content and psychometrically valid and appears to have interpretability to assess a threshold of MWPC for fatigue symptoms in this population. The modern psychometric evidence supports a unit-weighted total score (i.e., unweighted sum score) for the PROMIS-F SF-7a in this context, consistent with the development evidence disseminated by PROMIS [23, 52].

Fatigue is a meaningful and bothersome symptom of MAC lung disease, so it is important to be able to quantify the impact of fatigue on patients’ quality of life in clinical trials and clinical practice [9]. Patients consider fatigue to be one of the most significant symptoms affecting their daily life and are interested in interventions that can address fatigue [9]. This evidence validating the PROMIS-F SF-7a to assess fatigue in adult patients with MAC lung disease is therefore an important step in addressing the lack of validated PRO instruments in this population. Moreover, the PROMIS-F SF-7a is short (i.e., minimal respondent burden), quick to administer, and easy to understand and interpret by this population (based on previously completed qualitative research [19]), so it is well suited to use in clinical trials and clinical practice.

Floor effects were present for 3 items: ‘extreme exhaustion,’ ‘too tired to think clearly,’ and ‘too tired to bathe.’ However, it is logical that these items exhibited a tendency toward a likelihood of ‘never’ responses, as these fatigue manifestations would be expected to occur less frequently in patients with newly diagnosed MAC lung disease. In the previous qualitative research that confirmed the content validity of the PROMIS-F SF-7a in patients with MAC lung disease, approximately 22% and 38% of the cognitive interview participants indicated that the ‘too tired to think clearly’ and ‘too tired to bathe’ items, respectively, had not yet been a part of their experience with MAC [19]. The other response categories of ‘rarely’ and ‘sometimes’ were reasonably endorsed by patients, demonstrating the items’ utility in evaluating quality of life in the MAC lung disease population. Given the preponderance of evidence, there is no empirical support for item removal and the floor effects do not suggest reduced sensitivity in patients with mild fatigue. Similarly, although the ‘enough energy to exercise’ item had comparatively weaker inter-item correlations, the response categories of ‘rarely’ and ‘sometimes’ were reasonably endorsed by ARISE and ENCORE patients and the totality of evidence (e.g., good internal consistency) did not suggest a need for item removal.

The results of our psychometric validation of the PROMIS-F SF-7a in adults with an initial or subsequent diagnosis of MAC lung disease are similar to results reported in previous studies validating the instrument in different populations. As in our study, previous studies have found strong associations between the PROMIS-F SF-7a and other PRO instruments that measure fatigue, such as the Brief Fatigue Inventory or SF-36 vitality subscale [13, 53, 54]. Previous studies have also reported that the PROMIS-F SF-7a has strong internal consistency (Cronbach’s alpha > 0.70) in adults with chronic diseases such as HIV [55], ulcerative colitis [56], idiopathic inflammatory myopathies [57], rheumatoid arthritis [53], heart failure [54], sickle cell disease [13, 58], or fibromyalgia [13].

The estimated anchor-based threshold for clinically meaningful improvement in the PROMIS F-SF 7a score in our analysis was a reduction of 4 points from baseline, with a plausible range of −3 to −6 points from baseline for adults with an initial or subsequent diagnosis of MAC lung disease based on ARISE trial data. The eCDF curve for absolute change in PROMIS-F SF-7a score showed clear and consistent separation between PGI-S Fatigue change categories, with an estimated improvement threshold (−4 points, 95% CI: −3 to −6 points), further validating the anchor-based threshold. Our estimated MWPC is similar to meaningful change estimates proposed in previous studies in different populations, although it should be noted that MWPCs are condition-specific. In a study that used combined anchor- and distribution-based methods, the recommended minimally important difference range for the PROMIS-F SF-7a in patients with advanced-stage cancer was − 3 to −5 points [59]. In a study assessing the PROMIS-F SF-7a in patients with moderately to severely active inflammatory bowel disease using anchor-based methods, a 6-point and 6.4-point reduction in PROMIS-F SF-7a scores from baseline to Week 12 was identified as clinically meaningful in patients with Crohn’s disease and ulcerative colitis, respectively [56]. Although the MWPC threshold estimated in our analysis may not correspond to all patients’ experiences [26], the evidence supports its use in clinical trials to evaluate whether treatments for MAC lung disease meaningfully impact patients’ fatigue. It is also possible that our median MWPC threshold result (−4.00 points) could be used to interpret changes in and response to treatment in clinical practice, particularly once clinical trial data are available to inform treatment decision-making.

A strong association was observed between the PROMIS-F SF-7a and the FACIT-Fatigue (−0.80), which is another broadly used PRO instrument for fatigue. Although this was not a prespecified hypothesis, the result was in the expected direction and magnitude because greater convergent validity is expected in instruments that assess the same fatigue construct. Given the moderate-to-strong positive correlations observed between the PROMIS-F SF-7a and the SGRQ, EXACT, and E-RS, which are not limited to fatigue, future research should consider whether fatigue and respiratory symptoms are interdependent [51]. We speculate that fatigue may potentially be a distal outcome to culture conversion and respiratory symptom improvement; culture conversion is expected to occur while treatment for MAC is administered, subsequently leading to improved breathing as the infection clears. Improvement in fatigue, however, may follow biological and functional respiratory improvement due to the lengthy burden of MAC treatment. Consequently, studies with longer treatment duration (allowing for treatment tolerance and optimal biological response) and follow-up may be needed to observe the full impact of treatment on fatigue.

The psychometric validation analysis used data from geographically diverse, multi-center clinical trials, resulting in a large sample with follow-up data. Although the analysis sample was majority White, female, and aged ≥ 65 years, this is representative of adults with MAC lung disease [3, 4, 60]. One limitation is that the analysis did not include the presence and/or potential impacts of comorbidities, which may contribute to patients’ perceived fatigue and the responsiveness of fatigue to MAC-directed treatment. In addition, the test-retest interval was up to 70 days, which is longer than the ideal range of 2 to 3 weeks; however, longer retest intervals (e.g., 12 weeks) have been used in previous PROMIS-F SF-7a psychometric validation studies using clinical trial data [56]. Ideally, we could examine test-retest reliability in a subset of patients with a shorter retest interval, but this was not feasible given the small sample size. Therefore, although test-retest reliability was acceptable in this analysis and MAC lung disease progresses slowly, the results should be interpreted with caution. We did not find evidence of DIF in our analysis, but acknowledge that the sample sizes for the focal and reference groups were smaller than recommended (i.e., < 200; group samples that are < 200 are less effective at DIF detection) [17, 61], as well as the total number of items in the PROMIS-F SF-7a. There was very little missing data: only 1 patient was missing item-level PROMIS-F SF-7a data and no items demonstrated item response missingness.

The psychometric validation evidence supports the use of the PROMIS-F SF-7a score to accurately measure fatigue in adults with MAC lung disease. The PROMIS-F SF-7a can be considered for use to evaluate treatment response as an endpoint in MAC lung disease clinical trials. In combination with the results of the previous qualitative interviews, which were conducted in a real-world clinical practice setting and showed that fatigue is relevant to this patient population [19], the quantitative validation results suggest that PROMIS-F SF-7a could also be used to assess and monitor fatigue in patients with MAC lung disease in clinical practice. However, further research on monitoring meaningful improvement in fatigue specifically in the clinical practice setting is also warranted.

## Conclusions

The results of this psychometric validation analysis conducted in a large representative patient population demonstrate that the PROMIS-F SF-7a has adequate reliability, validity, and responsiveness for assessing fatigue symptoms in adults with a new or recurrent diagnosis of MAC lung disease. Fatigue, a critical manifestation of MAC lung disease, can now be assessed using the PROMIS-F SF-7a, a well-established, widely available, and easy to administer PRO instrument with well-understood measurement properties.

## Supplementary Information

Below is the link to the electronic supplementary material.

Supplementary Material 1

## Data Availability

The datasets generated and/or analysed during the current study are not publicly available due to containing confidential clinical trial data, but are available from the corresponding author on reasonable request.

## Abbreviations

ALIS: Amikacin liposome inhalation suspension
CFI: Comparative fit index
CI: Confidence interval
COA: clinical outcome assessment
DIF: Differential item functioning
eCDF: Empirical cumulative distribution function
ECV: Explained common variance
E-RS: EXACT Respiratory Symptoms
EXACT: Exacerbations of Chronic Pulmonary Disease Tool
FACIT: Functional Assessment of Chronic Illness Therapy
FDA: Food and Drug Administration
GRM: Graded response models
ICC: Intraclass correlation coefficient
iEFA: Item exploratory factor analysis
IRF: Item response function
IRT: Item response theory
MAC: *Mycobacterium avium* complex
MWPC: Meaningful within-patient change
NTM: Nontuberculous mycobacteria
PGI-S: Patient Global Impression of Severity
PRO: Patient-reported outcome
PROMIS-F SF-7a: Patient Reported Outcomes Measurement Information System Short Form v1.0 – Fatigue 7a
RMSEA: Root mean squared error of approximation
SGRQ: St. George Respiratory Questionnaire
TLI: Tucker-Lewis index

## Declarations

### Prior presentation

Part of this article has been presented at the ISPOR 2024 Conference, Atlanta, GA, May 5-8, 2024.
