# Supplementary material for: Psychometric validation of the PROMIS Fatigue-Short Form 7a in adults with newly diagnosed or recurrent Mycobacterium avium complex (MAC) lung disease: the ARISE and ENCORE studies
Source: J Patient Rep Outcomes. 2025 Oct 16;9:119. doi: 10.1186/s41687-025-00944-8 (PMC12532783; doi:10.1186/s41687-025-00944-8)
Supplement: Supplementary file 1 — Supplementary Material 1 [file 41687_2025_944_MOESM1_ESM.docx]

Supplementary Material

Supplementary Methods

Statistical Software

Item response patterns for the Patient Reported Outcomes Measurement Information System Short Form v1.0 – Fatigue 7a (PROMIS-F SF-7a) items were generated using the xtabs procedure in R [1]. Inter-item correlations were estimated using polychoric correlations obtained from version 2.3.3 of the psych package in R [2].

Item exploratory factor analyses, item response theory (IRT) models, local dependence, and differential item functioning (DIF) were evaluated using the mirt R package, version 1.39 [3].

For the internal consistency analysis, omega and alpha estimates were generated using the psych package, version 2.3.3 [2]; polyserial correlations were estimated with the polycor package, Version 0.8.1 [4]. The ICC[A,1] was estimated using the irr package, version 0.84.1 [5]. For convergent validity, Pearson correlations were estimated using the cor procedure embedded within base R [1]. The lm procedure embedded within base R [1], as well as the emmeans package, version 1.8.7 [6], and the effectsize package, version 0.8.3 [7], were used in the known-groups validity evaluation.

DIF Assessment

In the first stage of the Wald-2 DIF Sweep, the item parameter estimates were constrained to be the same across the groups, and the structural parameters (i.e., mean and variance of the latent distribution within the focal group) were estimated. A second IRT model was estimated in the first stage, in which the structural parameters for the focal group were held fixed at their previously estimated values, and the item parameters of both groups were freely estimated. The Wald test [8] with Benjamini-Hochberg false discovery rate (FDR) p-value adjustment was used to determine which items do not display DIF. The non-DIF items constituted the set of “anchor” items used in the second stage, and the remaining items were targets for formal DIF assessment in the second stage, defined next.

In the second stage of the procedure, the item parameters corresponding to the anchor items were held fixed across focal and reference groups, the structural parameters of the focal group were freely estimated, and the item parameters for the suspected DIF items were freely estimated across the focal and reference groups. By estimating the focal and reference group item parameters in this manner, the second stage of the DIF sweep is able to test whether item parameters significantly differ between groups adjusting for the structural parameter differences across groups. In so doing, the second stage tests for DIF while matching on latent score values—that is, given the same score, do the focal and reference groups systematically respond differently to a given item? By theory, they should not; if they do, then systematic item bias exists across groups. A second and final Wald test with a Benjamini-Hochberg FDR p-value adjustment was then used to determine which of the suspected DIF items, identified in the first stage, present with significant DIF after formal testing in the second stage. Any items that demonstrated significant DIF in the formal assessment of the second stage were scrutinized further to determine whether significant DIF was severe or not. This procedure is referred to as the DIF severity assessment.

In cases where no DIF exists, all items will present as “anchor” items in the first stage of the DIF-sweep, and the second stage is obviated because it is inestimable under this procedure. The conclusion in such an outcome is that no DIF exists. Further, no DIF severity assessment is possible in such a circumstance.

Item Elimination Protocol

Any evidence suggesting a need for alternative scoring or for item elimination was reviewed and decisions were only made after achieving consensus with the research team. Items were candidates for item removal if the team agreed that a preponderance of the following evidence pointed to a need to remove the items:

1. Items had sparse response categories (<10% endorsement or 0% endorsement in some categories);
2. Items demonstrated floor or ceiling effects;
3. Items had excessively weak (<0.2) or strong (>0.8) inter-item correlations;
4. Items did not clearly load on any estimated domain;
5. Items demonstrated uninterpretable (slope ~ 0; intercepts beyond + 3) or unstable (slope ~ + ∞; intercepts beyond + 3) item parameters, and had either a flat item response function or flat item information function;
6. Items demonstrated significant marginal χ2 and a Chen’s local dependence statistic exceeding values of 3;
7. Items demonstrated significant and/or meaningful DIF as measured by the weighted area between curves effect size;
8. Qualitative evidence corroborated the psychometric evidence;
9. Clinical knowledge corroborates the psychometric evidence.

Imputation

The handling of missing data and appropriate imputations were performed after all efforts failed to obtain the data. If appropriate, retrieved data were used. A retrieved assessment was defined as an assessment collected after the early discontinuation of amikacin liposome inhalation suspension (ALIS)/empty liposome control (ELC); a retriever was defined as a patient who stayed in the study and continued to provide data after discontinuation of the randomized treatment regimen.

Missing individual patient-reported outcome (PRO) items were not imputed. Missing PRO domain scores at Month 7 were imputed using the multiple imputation method, which is appropriate to estimate the mean and variance of the treatment effect.

The influence of intercurrent events (ICEs) and the presence of the missing data on the estimation of change from baseline to Month 7 PRO means were taken into consideration. The ICEs were defined as post-randomization events leading to early ALIS/ELC therapy termination due to death, ALIS/ELC-related treatment-emergent adverse event, use of rescue medication, or lack of efficacy.

The following considerations were included:

1. A patient who died after randomization would be assigned the worst change score in the entire sample as the change from baseline to Month 7
2. Data for patients who completed 6 months of the randomized treatment but their Month 7 PRO was not available were imputed based on the observed status distribution from the patients who completed 6 months of randomized treatment, had Month 7 data available, and were randomized to the same treatment group. This approach is consistent with a missing at random (MAR) approach.
3. When retrieved assessments were unavailable, the missing data were imputed as follows:

- For patients experiencing ICEs other than death, their missing Month 7 PRO data were imputed based on the retrieved assessment distribution (flagged as Ref_MNAR) from those retrievers who experienced ICE and are exposed to an altered treatment regimen after their ICEs. Missing not at random imputation of the data for those subjects experiencing ICE was applied.
- For patients not experiencing ICE, their missing Month 7 PRO data were imputed based on the observed data distribution (flagged as Ref_MNAR) of patients who completed 6 months of randomized therapy, had Month 7 PRO data assessment available, and were randomized to the same treatment group. This approach is consistent with a MAR approach.

The missing data were multiply imputed using PROC MI in SAS. Fifty replications were sufficient to reflect variability of the imputations. The variables used in the MI model (predictors) were randomization stratum (history of *Mycobacterium avium* complex lung infection, initial or subsequent), PRO score at baseline, and treatment group. The FCS option was used, allowing a joint distribution of continuous and categorical variables. To avoid imputed values being outside of range, the REGPREDMEANMATCH option was used. This method specifies the predictive mean matching method for continuous variables. It imputes a value randomly from a set of observed values whose predicted values are closest to the predicted value for the missing value from the specified model. The fixed seed, 68763497 (generated randomly), was used to enable reproducible results.

Following each round of imputation, a full set of PRO domain scores at Month 7 was created by combining the observed values and sets of imputed values under the MNAR and MAR assumptions. This process resulted in a total of 50 full sets. An analysis of covariance (ANCOVA) was performed using each of the 50 full sets. The ANCOVA model is described below. The 50 sets of results were then combined using Rubin’s rule implemented via SAS PROC MIANALYZE with the specification of their effect estimates together with the corresponding standard errors, resulting in a single set of estimates and testing statistics for the treatment effect.

ANCOVA model

The ANCOVA model included a response variable (change from baseline to Month 7) and the following independent variables: (1) history of *Mycobacterium avium* complex lung infection (initial or subsequent), (2) baseline PROMIS-F SF-7a value. The regression coefficient for treatment group in the ANCOVA was equal to the estimated population-level average treatment effect of μ_ALIS_−μ_ELC;_ this estimate was used in Wald tests deriving p-value for the difference between the treatment groups. The p-value was nominal as the study was not a hypothesis testing and further was not powered for this comparison.

The summaries included:

1. LS-mean of change from baseline to Month 7, differences of LS-mean changes from baseline (ALIS + background regimen minus ELC + background regimen) estimates and corresponding 95% CIs derived from ANCOVA model.
2. Descriptive statistics by timepoint with corresponding change from baseline.

Supplementary Tables

Supplementary Table 1. Principal ARISE and ENCORE Inclusion and Exclusion Criteria

| Inclusion criteria | - Male or female, ≥ 18 years of age (19 years or older in South Korea) - Current diagnosis of MAC lung infection. MAC or mixed infection with MAC as the dominant species is allowed, with MAC as the intended organism for treatment - Positive sputum culture for MAC within 6 months prior to screening - Positive sputum culture for MAC at Screening - A chest CT scan, read locally, within 6 months prior to Screening to determine presence and size of pulmonary cavities. Participants who do not have a chest CT scan within 6 months prior to Screening will be required to obtain a chest CT scan, read locally, during Screening - In the Investigator’s opinion, documented respiratory signs/symptoms at Screening that are attributable to the current MAC lung infection - An average QOL-B respiratory domain score of ≤ 85 based on scores at Screening and on the day of enrollment prior to randomization - In the Investigator’s opinion, underlying lung disease (e.g., COPD, bronchiectasis) has been managed according to best local standard of care, and on stable maintenance therapy for a minimum of 4 weeks prior to randomization - Willingness and ability to adhere to prescribed study treatment during the study - Ability to produce (spontaneously or with induction) approximately 2 mL of sputum for mycobacteriology at Screening - WOCBP (i.e., fertile following menarche and until becoming post-menopausal unless permanently sterile) and fertile men (i.e., all men after puberty unless permanently sterile by bilateral orchidectomy) agree to practice a highly effective method of birth control from Day 1 to at least 90 days after the last dose - Provide signed informed consent prior to administration of study drugs or performing any study related procedure - Be able to comply with study drugs use, study visits, and study procedures as defined by the protocol - Men with partners who are WOCBP (pregnant or non-pregnant) agree to use condoms and non-pregnant partners should practice a highly effective method of birth control |
| --- | --- |
| Exclusion criteria | - Diagnosis of cystic fibrosis - History of more than 3 MAC lung infections - Received any mycobacterial antibiotic treatment for current MAC lung infection - Refractory MAC lung infection, defined as having positive MAC cultures while being treated with a multidrug mycobacterial antibiotic treatment regimen for a minimum of 6 consecutive months and no documented successful treatment, defined as negative sputum culture for MAC and cessation of treatment - Relapse of prior MAC lung infection, defined as positive sputum culture for MAC ≤ 6 months of cessation of prior successful treatment - Evidence of any pulmonary cavity ≥ 2 cm in diameter, as determined by chest CT scan, read locally, within 6 months prior to Screening - Radiographic finding of new lobar consolidation, atelectasis, significant pleural effusion, or pneumothorax during routine clinical care within 2 months prior to Screening - Active pulmonary malignancy (primary or metastatic) or any malignancy requiring chemotherapy or radiation therapy within 1 year prior to Screening or anticipated during the study - Acute pulmonary exacerbation (e.g., COPD or bronchiectasis) requiring treatment with antibiotics, or corticosteroids (IV or oral), within 4 weeks prior to and during Screening - Current smoker - History of lung transplantation - Prior exposure to ALIS (including clinical study) - Known hypersensitivity or contraindications to use to ALIS, aminoglycosides, or any of their excipients - Disseminated MAC infection - Administration of any investigational drug within 8 weeks prior to Screening - Known or suspected acquired immunodeficiency syndromes (HIV-positive, regardless of CD4 counts). Other immunodeficiency syndromes that may interfere with study participation in the opinion of the Investigator - Current alcohol, medication, or illicit drug abuse - Known and active COVID-19 infection - Known hypersensitivity or contraindications to use to ethambutol, azithromycin (including other macrolides or ketolides), or any of their excipients per local labeling guidance |

Abbreviations: ALIS, amikacin liposome inhalation suspension; COPD, chronic obstructive pulmonary disease; CT, computed tomography; IV, intravenous; MAC, *Mycobacterium avium* complex; QOL-B, Quality of Life-Bronchiectasis; WOCBP, women of child-bearing potential

Supplementary Table 2. PROMIS-F SF-7a Item Response Pattern

| **PROMIS-F SF-7a Item** | **N (%) of Participants^a^** | | | | |
| --- | --- | --- | --- | --- | --- |
|  | **Never** | **Rarely** | **Sometimes** | **Often** | **Always** |
| How often did you feel tired? | 6 (2.61)^b^ | 20 (8.70)^b^ | 104 (45.22) | 79 (34.35) | 21 (9.13)^b^ |
| How often did you experience extreme exhaustion? | 56 (24.35)^c^ | 69 (30.00) | 75 (32.61) | 26 (11.30) | 4 (1.74)^b^ |
| How often did you run out of energy? | 27 (11.74) | 47 (20.43) | 106 (46.09) | 48 (20.87) | 2 (0.87)^b^ |
| How often did your fatigue limit you at work (including work at home)? | 40 (17.39) | 46 (20.00) | 90 (39.13) | 47 (20.43) | 7 (3.04)^b^ |
| How often were you too tired to think clearly? | 74 (32.17)^c^ | 65 (28.26) | 67 (29.13) | 24 (10.43) | 0 (0.00)^b^ |
| How often were you too tired to take a bath or shower? | 128 (55.65)^c^ | 52 (22.61) | 39 (16.96) | 9 (3.91)^b^ | 2 (0.87)^b^ |
| How often did you have enough energy to exercise strenuously? | 11 (4.78)^b^ | 37 (16.09) | 74 (32.17) | 64 (27.83) | 44 (19.13) |

^a^ No missing data. ^b^ Sparseness. ^c^ Floor effect.

Abbreviations: PROMIS-F SF-7a, Patient-Reported Outcomes Measurement Information System Short Form v1.0 – Fatigue 7a

Supplementary Table 3. PROMIS-F SF-7a Inter-Item Polychoric Correlations at Baseline

| **PROMIS-F SF-7a Item** | **Feel Tired** | **Extreme Exhaustion** | **Run Out of Energy** | **Fatigue Limit Work** | **Too Tired to Think** | **Too Tired to Bathe** | **Enough Energy to Exercise** |
| --- | --- | --- | --- | --- | --- | --- | --- |
| Feel Tired | 1.00 |  |  |  |  |  |  |
| Extreme Exhaustion | 0.70 | 1.00 |  |  |  |  |  |
| Run Out of Energy | 0.81 | 0.75 | 1.00 |  |  |  |  |
| Fatigue Limit Work | 0.72 | 0.74 | 0.77 | 1.00 |  |  |  |
| Too Tired to Think | 0.67 | 0.62 | 0.68 | 0.67 | 1.00 |  |  |
| Too Tired to Bathe | 0.63 | 0.58 | 0.59 | 0.58 | 0.71 | 1.00 |  |
| Enough Energy to Exercise | 0.27 | 0.13 | 0.25 | 0.28 | 0.22 | 0.23 | 1.00 |

Abbreviation: PROMIS-F SF-7a, Patient-Reported Outcomes Measurement Information System Short Form v1.0 – Fatigue 7a

Supplementary Table 4. Differential Item Functioning Group Characteristics

| **Characteristic** | **N (%)** |
| --- | --- |
| Sex |  |
| Male (reference) | 44 (19.1) |
| Female (focal) | 186 (80.9) |
| Age |  |
| Below median age (reference) | 122 (53.0) |
| Above median age (focal) | 108 (47.0) |
| MAC history |  |
| Initial (reference) | 178 (77.4) |
| Subsequent (focal) | 52 (22.6) |

Abbreviation: MAC, *Mycobacterium avium* complex.

Supplementary Table 5. Stage 1 DIF Sweep Results: Sex Split

| **PROMIS-F SF-7a Item** | **Parameter** | **Wald Statistic** | **Degrees of Freedom** | **Unadjusted P-Value** | **Benjamini-Hochberg Adjusted P-Value** |
| --- | --- | --- | --- | --- | --- |
| **Free intercept model** |  |  |  |  |  |
| Item 1 | D1, D2, D3, D4 | 3.51 | 4.00 | 0.48 | 0.72 |
| Item 2 |  | 5.41 | 4.00 | 0.25 | 0.58 |
| Item 3 |  | 5.56 | 4.00 | 0.23 | 0.58 |
| Item 4 |  | 4.99 | 4.00 | 0.29 | 0.58 |
| Item 6 |  | 0.63 | 4.00 | 0.96 | 0.96 |
| Item 7 |  | 0.73 | 4.00 | 0.95 | 0.96 |
| Item 1 | D1 | 0.67 | 1.00 | 0.41 | 0.92 |
| Item 2 |  | 1.08 | 1.00 | 0.30 | 0.92 |
| Item 3 |  | 0.01 | 1.00 | 0.92 | 0.92 |
| Item 4 |  | 0.09 | 1.00 | 0.77 | 0.92 |
| Item 5 |  | 0.78 | 1.00 | 0.38 | 0.92 |
| Item 6 |  | 0.18 | 1.00 | 0.67 | 0.92 |
| Item 7 |  | 0.06 | 1.00 | 0.81 | 0.92 |
| Item 1 | D2 | 0.58 | 1.00 | 0.45 | 0.66 |
| Item 2 |  | 1.08 | 1.00 | 0.30 | 0.66 |
| Item 3 |  | 1.52 | 1.00 | 0.22 | 0.66 |
| Item 4 |  | 1.03 | 1.00 | 0.31 | 0.66 |
| Item 5 |  | 0.15 | 1.00 | 0.70 | 0.81 |
| Item 6 |  | 0.52 | 1.00 | 0.47 | 0.66 |
| Item 7 |  | 0.01 | 1.00 | 0.94 | 0.94 |
| Item 1 | D3 | 1.33 | 1.00 | 0.25 | 0.44 |
| Item 2 |  | 2.32 | 1.00 | 0.13 | 0.44 |
| Item 3 |  | 1.60 | 1.00 | 0.21 | 0.44 |
| Item 4 |  | 2.20 | 1.00 | 0.14 | 0.44 |
| Item 5 |  | 0.30 | 1.00 | 0.58 | 0.63 |
| Item 6 |  | 0.29 | 1.00 | 0.59 | 0.63 |
| Item 7 |  | 0.24 | 1.00 | 0.63 | 0.63 |
| Item 1 | D4 | 0.82 | 1.00 | 0.37 | 0.84 |
| Item 2 |  | 0.07 | 1.00 | 0.79 | 0.84 |
| Item 3 |  | 1.15 | 1.00 | 0.28 | 0.84 |
| Item 4 |  | 0.04 | 1.00 | 0.84 | 0.84 |
| Item 6 |  | 0.04 | 1.00 | 0.84 | 0.84 |
| Item 7 |  | 0.57 | 1.00 | 0.45 | 0.84 |
| **Free slope model** |  |  |  |  |  |
| Item 1 | A1 | 1.35 | 1.00 | 0.25 | 0.75 |
| Item 2 |  | 0.00 | 1.00 | 0.96 | 0.98 |
| Item 3 |  | 0.17 | 1.00 | 0.68 | 0.96 |
| Item 4 |  | 2.45 | 1.00 | 0.12 | 0.75 |
| Item 5 |  | 0.00 | 1.00 | 0.98 | 0.98 |
| Item 6 |  | 0.63 | 1.00 | 0.43 | 0.75 |
| Item 7 |  | 0.71 | 1.00 | 0.40 | 0.75 |

Abbreviations: DIF, differential item functioning; PROMIS-F SF-7a, Patient-Reported Outcomes Measurement Information System Short Form v1.0 – Fatigue 7a.

Supplementary Table 6. Stage 1 DIF Sweep Results: Median Age Split

| **PROMIS-F SF-7a Item** | **Parameter** | **Wald Statistic** | **Degrees of Freedom** | **Unadjusted P-Value** | **Benjamini-Hochberg Adjusted P-Value** |
| --- | --- | --- | --- | --- | --- |
| **Free intercept model** |  |  |  |  |  |
| Item 1 | D1, D2, D3, D4 | 2.00 | 4.00 | 0.74 | 0.87 |
| Item 2 |  | 1.75 | 4.00 | 0.78 | 0.87 |
| Item 3 |  | 1.43 | 4.00 | 0.84 | 0.87 |
| Item 4 |  | 1.24 | 4.00 | 0.87 | 0.87 |
| Item 6 |  | 4.45 | 4.00 | 0.35 | 0.87 |
| Item 7 |  | 1.23 | 4.00 | 0.87 | 0.87 |
| Item 1 | D1 | 0.09 | 1.00 | 0.77 | 0.90 |
| Item 2 |  | 0.01 | 1.00 | 0.90 | 0.90 |
| Item 3 |  | 0.20 | 1.00 | 0.65 | 0.90 |
| Item 4 |  | 0.29 | 1.00 | 0.59 | 0.90 |
| Item 5 |  | 3.91 | 1.00 | 0.05 | 0.34 |
| Item 6 |  | 1.50 | 1.00 | 0.22 | 0.77 |
| Item 7 |  | 0.41 | 1.00 | 0.52 | 0.90 |
| Item 1 | D2 | 0.16 | 1.00 | 0.69 | 0.98 |
| Item 2 |  | 0.57 | 1.00 | 0.45 | 0.98 |
| Item 3 |  | 0.04 | 1.00 | 0.85 | 0.98 |
| Item 4 |  | 0.14 | 1.00 | 0.71 | 0.98 |
| Item 5 |  | 0.41 | 1.00 | 0.52 | 0.98 |
| Item 6 |  | 0.00 | 1.00 | 0.98 | 0.98 |
| Item 7 |  | 0.26 | 1.00 | 0.61 | 0.98 |
| Item 1 | D3 | 0.91 | 1.00 | 0.34 | 0.59 |
| Item 2 |  | 1.10 | 1.00 | 0.29 | 0.59 |
| Item 3 |  | 0.19 | 1.00 | 0.66 | 0.77 |
| Item 4 |  | 1.02 | 1.00 | 0.31 | 0.59 |
| Item 5 |  | 0.02 | 1.00 | 0.90 | 0.90 |
| Item 6 |  | 0.91 | 1.00 | 0.34 | 0.59 |
| Item 7 |  | 0.26 | 1.00 | 0.61 | 0.77 |
| Item 1 | D4 | 0.22 | 1.00 | 0.64 | 0.80 |
| Item 2 |  | 0.06 | 1.00 | 0.80 | 0.80 |
| Item 3 |  | 0.59 | 1.00 | 0.44 | 0.80 |
| Item 4 |  | 0.44 | 1.00 | 0.51 | 0.80 |
| Item 6 |  | 0.19 | 1.00 | 0.67 | 0.80 |
| Item 7 |  | 0.10 | 1.00 | 0.75 | 0.80 |
| **Free slope model** |  |  |  |  |  |
| Item 1 | A1 | 0.37 | 1.00 | 0.54 | 0.76 |
| Item 2 |  | 0.02 | 1.00 | 0.90 | 0.90 |
| Item 3 |  | 0.89 | 1.00 | 0.34 | 0.76 |
| Item 4 |  | 0.42 | 1.00 | 0.52 | 0.76 |
| Item 5 |  | 0.09 | 1.00 | 0.77 | 0.90 |
| Item 6 |  | 2.24 | 1.00 | 0.13 | 0.47 |
| Item 7 |  | 3.94 | 1.00 | 0.05 | 0.33 |

Abbreviations: DIF, differential item functioning; PROMIS-F SF-7a, Patient-Reported Outcomes Measurement Information System Short Form v1.0 – Fatigue 7a.

Supplementary Table 7. Stage 1 DIF Sweep Results: MAC History Split

| **PROMIS-F SF-7a Item** | **Parameter** | **Wald Statistic** | **Degrees of Freedom** | **Unadjusted P-Value** | **Benjamini-Hochberg Adjusted P-Value** |
| --- | --- | --- | --- | --- | --- |
| **Free intercept model** |  |  |  |  |  |
| Item 1 | D1, D2, D3, D4 | 3.61 | 4.00 | 0.46 | 0.74 |
| Item 2 |  | 3.05 | 4.00 | 0.55 | 0.74 |
| Item 3 |  | 1.90 | 4.00 | 0.75 | 0.75 |
| Item 4 |  | 2.66 | 4.00 | 0.62 | 0.74 |
| Item 6 |  | 4.00 | 4.00 | 0.41 | 0.74 |
| Item 7 |  | 6.96 | 4.00 | 0.14 | 0.74 |
| Item 1 | D1 | 0.15 | 1.00 | 0.70 | 0.80 |
| Item 2 |  | 0.83 | 1.00 | 0.36 | 0.64 |
| Item 3 |  | 0.08 | 1.00 | 0.77 | 0.80 |
| Item 4 |  | 0.07 | 1.00 | 0.80 | 0.80 |
| Item 5 |  | 1.03 | 1.00 | 0.31 | 0.64 |
| Item 6 |  | 1.20 | 1.00 | 0.27 | 0.64 |
| Item 7 |  | 2.54 | 1.00 | 0.11 | 0.64 |
| Item 1 | D2 | 0.23 | 1.00 | 0.63 | 0.82 |
| Item 2 |  | 0.23 | 1.00 | 0.63 | 0.82 |
| Item 3 |  | 0.18 | 1.00 | 0.67 | 0.82 |
| Item 4 |  | 0.02 | 1.00 | 0.90 | 0.90 |
| Item 5 |  | 0.52 | 1.00 | 0.47 | 0.82 |
| Item 6 |  | 0.14 | 1.00 | 0.71 | 0.82 |
| Item 7 |  | 3.71 | 1.00 | 0.05 | 0.38 |
| Item 1 | D3 | 1.52 | 1.00 | 0.22 | 0.38 |
| Item 2 |  | 0.61 | 1.00 | 0.44 | 0.61 |
| Item 3 |  | 1.77 | 1.00 | 0.18 | 0.38 |
| Item 4 |  | 0.00 | 1.00 | 0.96 | 0.96 |
| Item 5 |  | 0.01 | 1.00 | 0.92 | 0.96 |
| Item 6 |  | 2.86 | 1.00 | 0.09 | 0.32 |
| Item 7 |  | 4.50 | 1.00 | 0.03 | 0.24 |
| Item 1 | D4 | 0.29 | 1.00 | 0.59 | 0.93 |
| Item 2 |  | 0.12 | 1.00 | 0.73 | 0.93 |
| Item 3 |  | 0.00 | 1.00 | 0.95 | 0.95 |
| Item 4 |  | 2.34 | 1.00 | 0.13 | 0.76 |
| Item 6 |  | 0.08 | 1.00 | 0.78 | 0.93 |
| Item 7 |  | 0.27 | 1.00 | 0.60 | 0.93 |
| **Free slope model** |  |  |  |  |  |
| Item 1 | A1 | 0.37 | 1.00 | 0.54 | 0.76 |
| Item 2 |  | 0.02 | 1.00 | 0.90 | 0.90 |
| Item 3 |  | 0.89 | 1.00 | 0.34 | 0.76 |
| Item 4 |  | 0.42 | 1.00 | 0.52 | 0.76 |
| Item 5 |  | 0.09 | 1.00 | 0.77 | 0.90 |
| Item 6 |  | 2.24 | 1.00 | 0.13 | 0.47 |
| Item 7 |  | 3.94 | 1.00 | 0.05 | 0.33 |

Abbreviations: DIF, differential item functioning; PROMIS-F SF-7a, Patient-Reported Outcomes Measurement Information System Short Form v1.0 – Fatigue 7a.

References

1. R Core Team. R: A Language and Environment for Statistical Computing. R Foundation for Statistical Computing, Vienna. 2023. https://www.R-project.org

2. Revelle W. Psych: Procedures for Psychological, Psychometric, and Personality Research. R package version 2.3.6. Northwestern University, Evanston, Illinois. https://CRAN.R-project.org/package=psych.

3. Chalmers RP. mirt: a multidimensional item response theory package for the R environment. J Stat Softw. 2012;48(6):1-29. doi:10.18637/jss.v048.i06.

4. Fox J. polycor: Polychoric and Polyserial Correlations. R package. 2022;Version 0.8.1. 2022. https://cran.r-project.org/web/packages/polycor/polycor.pdf.

5. Gamer M, Lemon J, Singh I. irr: Various Coefficients of Interrater Reliability and Agreement. Version 0.84.1. 2010. https://cran.r-project.org/web/packages/irr/irr.pdf.

6. Lenth R. emmeans: Estimated Marginal Means, aka Least-Squares Means. R package 2023;Version 1.8.7. https://cran.r-project.org/web/packages/emmeans/emmeans.pdf.

7. Ben-Shachar MS, Lüdecke D, Makowski D. effectsize: Estimation of effect size indices and standardized parameters. J Open Source Softw. 2020;5(56):2815. doi:10.21105/joss.02815.

8. Wald A. Tests of statistical hypotheses concerning several parameters when the number of observations is large. Trans Am Math Soc. 1943;54(3):426-82.
